# *ANKH *variants associated with ankylosing spondylitis: gender differences

**DOI:** 10.1186/ar1701

**Published:** 2005-02-25

**Authors:** Hing Wo Tsui, Robert D Inman, Andrew D Paterson, John D Reveille, Florence WL Tsui

**Affiliations:** 1 Toronto Western Research Institute, Toronto, Ontario, Canada; 2 University of Toronto, Toronto, Ontario, Canada; 3 The Hospital for Sick Children, Toronto, Ontario, Canada; 4 The University of Texas-Houston Health Science Center, Houston, Texas, USA

## Abstract

The *ank *(progressive ankylosis) mutant mouse, which has a nonsense mutation in exon 12 of the inorganic pyrophosphate regulator gene (*ank*), exhibits aberrant joint ankylosis similar to human ankylosing spondylitis (AS). We previously performed family-based association analyses of 124 Caucasian AS families and showed that novel genetic markers in the 5' flanking region of *ANKH *(the human homolog of the murine *ank *gene) are modestly associated with AS. The objective of the present study was to conduct a more extensive evaluation of *ANKH *variants that are significantly associated with AS and to determine whether the association is gender specific. We genotyped 201 multiplex AS families with nine *ANKH *intragenetic and two flanking microsatellite markers, and performed family-based association analyses. We showed that *ANKH *variants located in two different regions of the *ANKH *gene were associated with AS. Results of haplotype analyses indicated that, after Bonferroni correction, the haplotype combination of *rs26307 [C] *and *rs27356 [C] *is significantly associated with AS in men (recessive/dominant model; *P *= 0.004), and the haplotype combination of *rs28006 [C] *and *rs25957 [C] *is significantly associated with AS in women (recessive/dominant model; *P *= 0.004). A test of interaction identified *rs26307 *(i.e. the region that was associated in men with AS) as showing a difference in the strength of the association by gender. The region associated with AS in women only showed significance in the test of interaction among the subset of families with affected individuals of both genders. These findings support the concept that *ANKH *plays a role in genetic susceptibility to AS and reveals a gender–genotype specificity in this interaction.

## Introduction

Ankylosing spondylitis (AS) is a disorder that results in chronic joint and entheseal inflammation, and ankylosis of axial and peripheral joints. It affects approximately 0.1–0.8% of Caucasians [1]. The disease usually begins in young adulthood and can be associated with chronic pain and significant disability. AS is strongly associated with HLA-B27 [2], but analyses of recurrence risk among family members [3] suggest that at least three other genetic loci in addition to HLA-B27 are required to confer full susceptibility to AS. However, genome-wide linkage studies have detected very few strongly linked non-major histocompatibility complex (MHC) loci [4-6], implying that non-MHC susceptibility loci have small effects and/or that heterogeneous sets of loci combine with HLA-B27 to confer susceptibility to AS. This complexity highlights the strategic advantage of testing predetermined candidate genes. In addition, although several chromosomal regions showed potential linkage in several genome-wide linkage studies conducted in AS families [5,6], the identities of the predisposing genes in these regions remain largely unknown.

Normal osteogenesis depends critically on maintaining the physiological level of inorganic pyrophosphate (PPi). Abnormal PPi levels can be associated with aberrant bone formation. PPi export from the cell is regulated by the ANK protein [7], and mutant mice (*ank/ank*), which have a premature stop codon in the 3' end of the *ank *gene, develop severe ankylosis. As a first step in testing the hypothesis that specific polymorphisms in the *ANKH *gene might contribute to AS susceptibility, we previously reported the identification of two novel polymorphic sites, one in the 5' noncoding region (*ANKH-OR*) and the other in the promoter region (*ANKH-TR*), of *ANKH *[8]. These two marker alleles are in complete linkage disequilibrium (LD). Our results from a linkage analysis of 124 North American AS families [8] indicated that AS is genetically linked to *ANKH*, and the locus-specific sibling recurrence risk of *ANKH *to AS susceptibility (λ_S_) is 1.9 (λ_S _for HLA-B27 is 5.2). Our family-based association analysis on the same families [8] showed that AS is modestly associated with *ANKH-OR *allele 1 (additive model: *P *= 0.03). Because of insufficient numbers of informative families, our results did not allow us to distinguish between different modes of inheritance. In addition, our analyses were focused on the 5' end of the gene, using only two markers. For these reasons, we have now carried out fine mapping of the complete *ANKH *region, including not only the AS families used in the previous study but also an additional 77 multiplex AS families (a total of 201 multiplex AS families).

The prevalence of AS is 2.5 times higher in men than in women [9]. Extensive fusion of the spine is a phenotype of the mouse model *ank*. There has been a clinical impression that radiographic severity (e.g. the bamboo spine) may be relatively less common in affected women than in men [10-13]. It has also been observed that long-term outcome in AS is worse in men than in women [14,15], but the basis for this difference in severity of clinical expression remains unclear. It is unlikely that the major genetic factors that account for these differences are X-linked because there is no linkage of AS susceptibility with X-chromosome markers [16]. Gender also has a significant impact on heritability in AS. AS has a higher prevalence in the offspring of women than men with AS, and sons of men with AS are 2.5 times more likely than daughters to inherit the disease [17,18]. It remains unclear whether there is gender heterogeneity in non-MHC loci that confer susceptibility to AS. In the present study, we asked whether there is any gender difference in the association of *ANKH *with AS in multiplex families.

## Materials and methods

### Ankylosing spondylitis families

The study group comprised 201 Caucasian AS families (a total of 226 nuclear families; Tables 1 and 2). This group was recruited from the Toronto Western Spondylitis Clinic (23 families) and from other sites in the North American Spondylitis Consortium (178 families). All patients met modified New York criteria for the diagnosis of AS [19], which include radiographic evidence of sacroiliitis. Of the affected and unaffected individuals, 60% and 47% were men, respectively. The ages of the individuals ranged from 8 to 75 years. The study was approved by the University Health Network Research Ethics Board and the Committee for the Protection of Human Subjects at the University of Texas Health Science Center-Houston.

### Genotyping

DNA from the affected and unaffected family members was prepared from peripheral blood lymphocytes using standard techniques.

#### Microsatellite markers

Genotyping was performed using three microsatellite markers flanking *ANKH *on chromosome 5p: *D5S1953*, *D5S1991 *and *D5S1954*. Polymerase chain reaction fragments were run on native polyacrylamide gel, stained with ethidium bromide and visualized using an imager (Bio-Rad, Hercules, CA, USA).

#### Single nucleotide polymorphisms

Genotyping was performed using seven intronic single nucleotide polymorphisms (SNPs; *rs26307 [C/T]*, *rs27356 [C/T]*, *3088132 [G/C]*, *rs153929 [A/G]*, *rs258215 [A/T]*, *rs28006 [C/T] *and *rs25957 [C/G]*). Optimized allelic discrimination assays for SNPs were purchased from Applied Biosystems (Foster City, CA, USA). The plates were read on an ABI PRISM 7900 sequence detection system (Applied Biosystems).

### Statistical analysis

#### Error checking

To minimize data errors, extensive error checking procedures were used. For microsatellite markers, allele assignment was checked manually for all genotypes by two independent individuals. Size data were converted into discrete allele numbers; samples not following Mendelian patterns of inheritance were identified using Pedmanager (available online at ), and these samples were subjected to repeat genotyping.

#### Family-based association analyses

The transmission disequilibrium test (TDT) was used to test for transmission of specific alleles from heterozygous parents to affected offspring [20]. We computed the test statistics using the empirical variance option of family-based association testing (FBAT) software, version 1.5.5 (available online at ) [21]. This option is used when testing for associations in an area of known linkage (the null hypothesis assumes no association but linkage) with multiple affected siblings in a family or when multiple nuclear families in a pedigree are considered. This program uses data from nuclear families, sibships, pedigrees or any combination, and provides unbiased tests with or without founder genotypes. Biallelic tests were performed using additive, dominant/recessive genetic models. Haplotype analyses were carried out using the haplotype-based association testing (HBAT) empirical variance (-e) option in the FBAT program. For Bonferroni correction, because eight tests (four haplotypes and two models) were carried out in the HBAT-e analyses, *P *< 0.00625 (0.05/8) was considered statistically significant.

For analysis of affected men/women, the FBAT command 'setafftrait' was used. The unaffected siblings and parents from the families were coded as unknown (0) phenotype, the affected men were coded as 2, and the affected women as 1. FBAT-e analyses using the setafftrait 1 0 0 command were used to test specifically for affected men, and analyses using the setafftrait 0 -1 0 command were used to test specifically for affected women. To test for differences between family-based association for affected men and women, the setafftrait 1 -1 0 command was used.

TDT was used to estimate the frequency of transmission to the affected men or women of the haplotypes of interest. Findings in one affected individual, randomly selected from each of the multiplex families, were used in the calculations.

## Results

### Association between specific *ANKH *variants and ankylosing spondilitis

**Figure 1:**
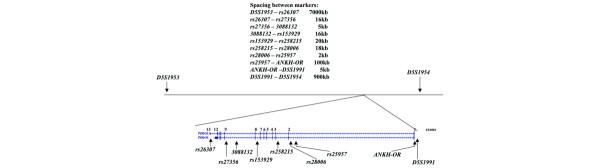
Locations and spacings of genetic markers used for genotyping. *D5S1991 *and *ANKH-OR *are located at the 5' flanking region of *ANKH*. All seven single nucleotide polymorphisms used are located in the introns of *ANKH*.

The *ANKH *gene encodes for *ANKH *transcripts with different lengths at the 3' untranslated region. The longer transcript (3928 bp; AB046801) is derived from 12 exons, whereas the shorter transcript (2426 bp; AK001799, which contains the last 1721 bp of this transcript) is derived from 13 exons. We fine-mapped the *ANKH *gene using 11 markers (Fig. 1): three microsatellite markers (*D5S1954*, *D5S1991 *and *D5S1953*), one 5' untranslated region variant (*ANKH-OR*), and seven intronic SNPs (*rs25957 *and *rs28006 *in intron 1, *rs258215 *in intron 2, *rs153929 *in intron 7, *3088132 *and *rs27356 *in intron 8, and *rs26307 *in intron 12).

As an extension to our previous study [8], we included a total of 201 multiplex AS families in a family-based association analysis (77 additional multiplex AS families were included, in addition to the 124 AS families considered in the first study). All of the families were genotyped with 11 markers in the *ANKH *region (*D5S1953*, *rs26307*, *rs27356*, *3088132*, *rs153929*, *rs258215*, *rs28006*, *rs25957*, *ANKH-OR*, *D5S1991 *and *D5S1954*). FBAT analyses showed two regions in the *ANKH *gene where associations between *ANKH *variants and AS were detected. Using both additive and recessive models, *rs27356 [C] *was significantly associated with AS (additive model: Z score = 2.54, *P *= 0.011; recessive model: Z score = 2.32, *P *= 0.020). However, depending on the model used for the analysis, two different *ANKH *markers were also associated with AS. Using an additive model, an intron 1 SNP, namely *rs25957 [C]*, was associated with AS (Z score = 2.02, *P *= 0.043). Using a dominant model, *ANKH-OR *allele 1 was associated with AS (Z score = 2.20, *P *= 0.027). The results are summarized in Table 3. However, these markers are located in different haplotype or LD blocks (see below), implying that there is more than one susceptibility locus in the *ANKH *gene.

Thus, our analyses of 201 multiplex AS families showed that *ANKH *variants found in two different regions of the *ANKH *gene are modestly associated with AS. Our working hypothesis was that there are two subsets of AS patients, each with a different predisposing polymorphism in the *ANKH *locus. Because *ANKH *has been shown to be an androgen responsive gene [22-24], we considered whether there are gender differences between family-based associations of *ANKH *variants to AS.

### Men with ankylosing spondilitis differ from affected women for association with different *ANKH *variants

**Figure 2:**
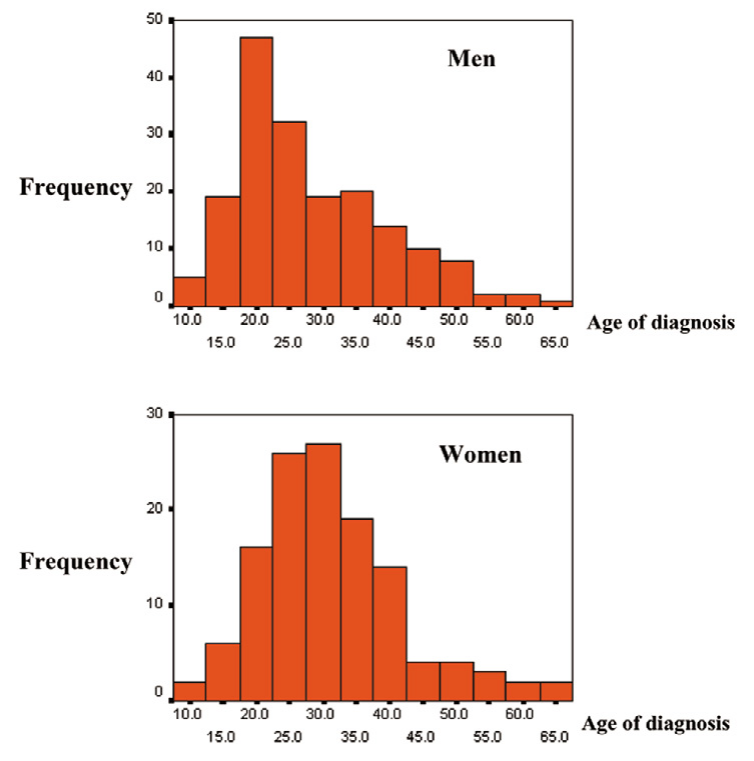
Age of diagnosis for **(a) **men and **(b) **women in the North American multiplex ankylosing spondilitis families.

Radiographic features of AS vary between men and women, with extensive spinal ankylosis being relatively infrequent in women with AS [10]. Table 2 summarizes gender information for the affected individuals in the 201 North American multiplex AS families. There were 94 families with both affected men and women in each family, 74 families with affected men only, and 33 families with affected women only. In this cohort of North American multiplex AS families, men have a significantly earlier age at diagnosis than that for women (mean [± standard deviation] age of diagnosis for affected men = 28 ± 11 years [*n *= 213]; mean age of diagnosis for affected women = 30 ± 11 years [*n *= 149]; including family as an independent variable [using SAS PROC GLM; SAS Institute Inc., Cary, NC, USA]: F = 5.10, *P *= 0.025; Fig. 2). In addition, analysis of age at AS diagnosis in affected men did not reveal a normal distribution; rather the distribution was skewed toward an earlier onset.

In view of these gender differences, we re-analyzed our genotyping results along gender lines in two separate FBAT analyses using the setafftrait command. FBAT analysis of transmission of alleles to affected women showed that both *rs25957 [G] *and *rs28006 [T] *were associated with AS (additive model and biallelic test: *rs25957 [G]*, Z score = 2.82, *P *= 0.004; *rs28006 [T]*, Z score = 2.82, *P *= 0.004; Table 4). These results indicate that only *ANKH *variants at the 5' end, and not those at the 3' end, of *ANKH *are associated with AS in affected women. This also suggested that *ANKH *variants at the 3' end of the gene might be associated with AS only in affected men.

To test this hypothesis, we analyzed transmission of alleles to affected men. FBAT analysis of transmission of alleles to affected men using the setafftrait command showed that two neighbouring *ANKH *variants at the 3' end of the gene, namely *rs26307 [C] *and *rs27356 [C] *(16 kb apart), were associated with AS in affected men as was predicted (additive model: *rs26307 [C]*, Z score = 2.06, *P *= 0.039; *rs27356 [C]*, Z score = 2.63, *P *= 0.008; recessive model: *rs26307 [C]*, Z score = 2.51, *P *= 0.012; *rs27356 [C]*, Z score = 2.99, *P *= 0.002; Table 5).

### Identification of *ANKH *haplotypes that are associated with ankylosinig spondylitis

Where the aetiological variant is not typed, haplotype-based analysis is more powerful for association studies in which there is significant LD in the region of interest. We took advantage of the data from the HapMap project (12 October 2004 release 12; [25]). The markers we used for genotyping are located in four different haplotype blocks (block 1: *rs26307*, *rs27356*; block 2: *3088132 *and *rs153929*; block 3: *rs28006 *and *rs25957*; block 4: *ANKH-OR *and *D5S1991*).

We carried out haplotype analyses based on this information, using the HBAT empirical variance option in the FBAT program, and the results are summarized in Table 6. For HBAT analyses considering all 226 AS nuclear families, in each of three different haplotype blocks (blocks 1, 2 and 4) there was one haplotype with a significant *P *value, suggesting that there is heterogeneity in this locus. When HBAT analyses were carried out specifically for affected women, a haplotype with a significant *P *value was found in haplotype block 3 located at the 5' end of the gene. When HBAT analyses were conducted specifically for affected men, one haplotype with a significant *P *value was present in block 1, which is located at the 3' end of the gene. These results are consistent with those from single-marker tests in the FBAT analyses. Furthermore, after Bonferroni correction for the number of haplotypes and models (*n *= 8), the haplotype combination of *rs26307 [C] *and *rs27356 [C] *remained significantly associated with AS in men (recessive/dominant model: *P *= 0.004), and the haplotype combination of *rs28006 [C] *and *rs25957 [C] *was significantly associated with AS in women (recessive/dominant model: *P *= 0.004).

### A direct test for differences between family-based association with affected men and women

In order to conclude that there are gender differences in *ANKH *variants associated with AS, one must show significant heterogeneity between affected men and women. For this purpose, we used the setafftrait 1 -1 0 command to conduct the FBAT-e analyses. We coded unaffected siblings and parents from the families as unknown phenotype (0), affected men as phenotype 2, and affected women as phenotype 1. The setafftrait 1 -1 0 command converted affect status to trait 1 (affected men), -1 (affected women) and 0 (unaffected siblings and parents), and the results are summarized in Table 7. The only marker with a significant *P *value was *rs26307 [C] *(dominant/recessive model: *P *= 0.03), suggesting that this marker was significantly associated with AS only in affected men.

In view of this finding, we considered whether there is a subset of AS multiplex families in which *ANKH *variants were significantly associated with AS only in affected women. As summarized in Table 2, there were two types of families in our cohort of multiplex AS families: families with affected individuals of both genders; and families with only one gender of affected individuals (either affected men or affected women).

To assess whether there was significant heterogeneity between affected men and women in the families of the first family type (with affected men and women in each family), we used the setafftrait 1 -1 0 command to conduct the FBAT-e analyses. The results are summarized in Table 8. Two markers (*rs28006 [T] *and *rs25957 [G]*) exhibited significant *P *values (additive model: *P *= 0.004 for *rs28006 *and *P *= 0.017 for *rs25957*), suggesting that these two markers were associated with AS only in affected women in the subset of AS families with affected individuals of both genders.

We also conducted FBAT-e analysis using setafftrait command 1 -1 0 in families with only one gender of affected individuals (data not shown). However, there were few informative families (<20 families from which we could track the transmission of alleles), and so the results might not be reliable.

### Selective transmission of haplotypes of interest to the affected men/women

In order to estimate the magnitude of the effect, we calculated the frequency at which the haplotypes of interest were transmitted to the affected men or women using TDT. For the haplotype *rs28006 [C] rs25957 [C]*, the frequency of transmission was 74% (17/23) to affected women and 40% (12/30) to affected men. Thus, the 'odds ratio' for increased risk is 1.85 (0.74/0.4). More dramatic proportions were seen in the subset of families with affected individuals of both genders. In these families, this haplotype was transmitted to affected women 79% of the time (15/19) but to affected men only 27% of the time (3/11). In this case, the 'odds ratio' for increased risk approaches 3.0 (0.79/0.27 = 2.92).

For the haplotype *rs26307 [C] rs27356 [C]*, the frequency of transmission was 70% (21/30) to affected men and 43% (13/30) to affected women (an 'odds ratio' for increased risk of 1.75). In the subset of families with only affected men, 94% (16/17) of the time this haplotype was transmitted to affected men. There were too few informative families with only affected women with this variant (*n *= 6), and so we do not have a reliable assessment of the frequency at which this haplotype was transmitted to affected women in this subset for comparison.

## Discussion

In this study of the association of *ANKH *genetic markers with AS, including 201 AS multiplex families, we found that *ANKH *variants located in two different regions of the *ANKH *gene were associated with AS. A more striking finding was that the genetic association for men and women with AS differed. In men, AS was associated with genetic markers at the 3' end of the *ANKH *gene, whereas in women AS appeared to be associated with genetic markers at the 5' end of the *ANKH *gene. As expected, when the genders of AS patients were analyzed separately, we observed more than one SNP in each region (within the same haplotype block) showing significant association with AS. Haplotype analyses appeared to confirm the results of the single-marker tests (FBAT analyses), indicating that the predisposing polymorphism(s) for men with AS probably lies at the 3' end of the *ANKH *gene, whereas those for affected women are probably at the 5' end of the gene. After Bonferroni correction for the number of haplotypes and models, the haplotype combination of *rs26307 [C] *and *rs27356 [C] *was significantly associated with men with AS; and the haplotype combination of *rs28006 [C] *and *rs25957 [C] *was significantly associated with women with AS. However, in both cases, the significance level was modest. We attribute this to the fact that we have not identified the aetiological variants in the men/women with AS. Despite the modest *P *values (which are a function of sample size), the calculated 'odds ratios' for increased risk (which provide estimates of the magnitude of the effect) were close to 2 for the transmission of *rs26307 [C] rs27356 [C*] to affected men, and close to 3 for the transmission of *rs28006 [C] rs25957 [C] *to affected women in the subset of families with affected individuals of both genders.

A test of interaction identified the region that was associated in men with AS (*rs26307*) as showing a difference in the strength of the association by gender. The region associated with AS in women only showed significance of the test of interaction among the subset of families with affected individuals of both genders. Our current efforts are to identify and analyze more common SNPs in these two regions, ultimately finding the predisposing polymorphisms in men and women.

The rationale for studying multiplex AS families is to enhance the chances of identifying the genes involved. There are very few studies that directly compare familial versus sporadic AS. In one study [26], familial versus sporadic Dutch AS patients exhibited no difference in age at disease onset, age at diagnosis, or prevalence of peripheral arthritis and acute anterior uveitis. In another study, familial AS disease was significantly milder than sporadic disease, as assessed by spinal mobility score, Arthritis Impact Measurement Scales (AIMS) overall impact score, AIMS physical activity score, AIMS social function score and AIMS pain score [27]. Thus, findings from multiplex families might not be directly applicable to individuals affected with sporadic AS. Most studies assessing the impact gender has on age at AS onset or diagnosis have been conducted without addressing whether the individuals had familial or sporadic disease [28-30]; these studies showed that the age at disease onset is similar between genders. However, in our cohort of AS multiplex families, men had a significantly earlier age at diagnosis compared with that for women (for men 28 ± 11 years [*n *= 213]; and for women 30 ± 11 years [*n *= 149]). Because these are AS multiplex families, it is unlikely that there is a bias leading physicians to delay diagnosis in affected women. The misconception that AS is exclusively a male disease may yet be a confounding factor. In the subset of families with affected individuals of both genders, men have an even earlier age at diagnosis (27.8 ± 11 years [*n *= 94]) compared with that for women (32.6 ± 11 years [*n *= 101]), whereas both men and women have similar ages of diagnosis in the subsets of families with affected individuals of only one gender (for men 28.6 ± 12 years [*n *= 130]; for women 29.9 ± 12 years [*n *= 53]). This finding suggests that there is heterogeneity even in multiplex AS families.

The *ANKH *variants that were significantly associated with AS are located in introns 1, 8 and 12. It is likely that the predisposing polymorphisms affect gender-specific regulation of *ANKH *expression. Very little is known regarding the molecular mechanisms that underlie the regulation of *ANKH *expression. One study [31] reported that *ANKH *is a growth factor responsive gene. Three recent reports [22-24] showed that *ANKH *is an androgen responsive gene. In androgen-treated prostate cancer cell lines, the abundance of *ANKH *transcripts was sixfold higher than in the untreated cells. In the *ANKH *promoter, there is a sequence at position -1015 (AGAACAcacTtTcCT) with 83% match to an androgen response element (ARE) consensus sequence [22]. It remains unclear whether this ARE-like motif is functional. In view of the locations of the *ANKH *variants associated with AS, it remains unclear whether this ARE-like motif at the promoter region can directly contribute to the regulation of *ANKH *expression by the predisposing polymorphisms. It is also unknown whether there is a different mode of *ANKH *regulation in women.

A report recently concluded that *ANKH *did not significantly contribute to susceptibility or specific disease expression in AS patients from the UK [32]. In that report, a case–control study was conducted using five *ANKH *SNPs within the coding region and flanking splice sites and three known promoter variants. There was no association between these polymorphisms and AS or the clinical pattern of the disease. In addition, using 185 affected sib-pair AS families, no linkage between *ANKH *and AS was observed. However, the exact linkage results were not shown. Using multipoint exclusion mapping of the *ANKH *region, the presence of a gene contributing more than 10% of the recurrence risk to AS (λ_S _= 1.4) was excluded. Using λ_S _of 1.4 as the cutoff may exclude genes with modest effects. In that report, the LD between markers was not shown. In situations where the aetiological variant is not typed, haplotype-based analysis may be a more powerful analytical method when there is significant LD.

The basis for the discrepancy between the UK results [32] and ours is not entirely clear, but there are several possible explanations. First, the UK group focused on analyzing exonic variants, variants near splice junctions and in the promoter region. Their analysis did not include any *ANKH *variants in the 3' region, where we detected association with AS in men. Second, although the UK group included a gender breakdown of their patients (63.5% men and 36.5% women), the analysis did not include a breakdown of AS patients by gender, and variants with modest gender-specific effects might have been missed. Third, it is possible that there are some intrinsic differences between the two populations (UK versus North American). Genome-wide linkage scans performed in the two groups revealed some similar susceptibility regions, such as on chromosomes 6p (the MHC), 5q and 10q [5,6]. However, the linkage identified on chromosome 11q23 in the North American Spondylitis Consortium families was not seen in the UK study. In addition, the linkage identified on chromosome 2q in the UK study was not seen in the North American Spondylitis Consortium study. The intrinsic differences could reflect clinical differences in the patient population recruited, or they could be due to population-specific mechanisms of genetic susceptibility. Finally, because both groups analyzed about 200 AS families, there might not be sufficient power to detect genes with 'small effects' consistently, leading to discrepancies between results.

In our cohort of North American multiplex AS families, the age at diagnosis was significantly younger in men than in women. However, FBAT analyses using the offset option (-o; an option which works for both quantitative and qualitative traits) did not show any significant association of age at diagnosis in the men or women with AS, even in subsets of families, using the *ANKH *markers (data not shown), suggesting that *ANKH *variants are responsible for disease susceptibility. Our finding of gender-specific polymorphisms in the *ANKH *gene conferring differential susceptibility to AS might shed light on the biological basis of these clinical observations.

In view of the difficulty in locating susceptibility loci with modest effects in recent genome-wide linkage studies conducted in AS families, it will be of interest to assess whether gender subsetting in the analyses of genome-wide linkage studies might yield further insights into the genetic basis of rheumatic diseases, many of which have a strong gender predilection.

## Conclusion

Taken together, our findings showed that, after Bonferroni correction, two intronic markers at the 3' end of the *ANKH *gene were significantly associated with AS only in affected men, and two intronic markers at the 5' end of the *ANKH *gene were significantly associated with AS only in affected women. This may partly account for the gender difference in the prevalence of AS.

## Abbreviations

AIMS = Arthritis Impact Measurement Scales; ARE = androgen response element; AS = ankylosing spondylitis; bp = base pairs; FBAT = family-based association testing; HBAT = haplotype-based association testing; LD = linkage disequilibrium; MHC = major histocompatibility complex; PPi = inorganic pyrophosphate; SNP = single nucleotide polymorphism; TDT = transmission disequilibrium test.

## Competing interests

The author(s) declare that they have no competing interests.

## Authors' contributions

HWT conducted all of the genotyping and analyzed the data. RDI conceived the study, provided some of the patients' blood/cells for extracting DNA and reviewed the manuscript. ADP designed the study, supervised the statistical analyses and revised the manuscript. JDR coordinated the recruitment of individuals from AS families, provided most of the DNA samples and reviewed the manuscript. FWLT conceived, designed and coordinated the study, analyzed and interpreted the data, performed statistical analyses, and drafted and revised the manuscript. All authors read and approved the final manuscript.

## Figures and Tables

**Figure 3:**
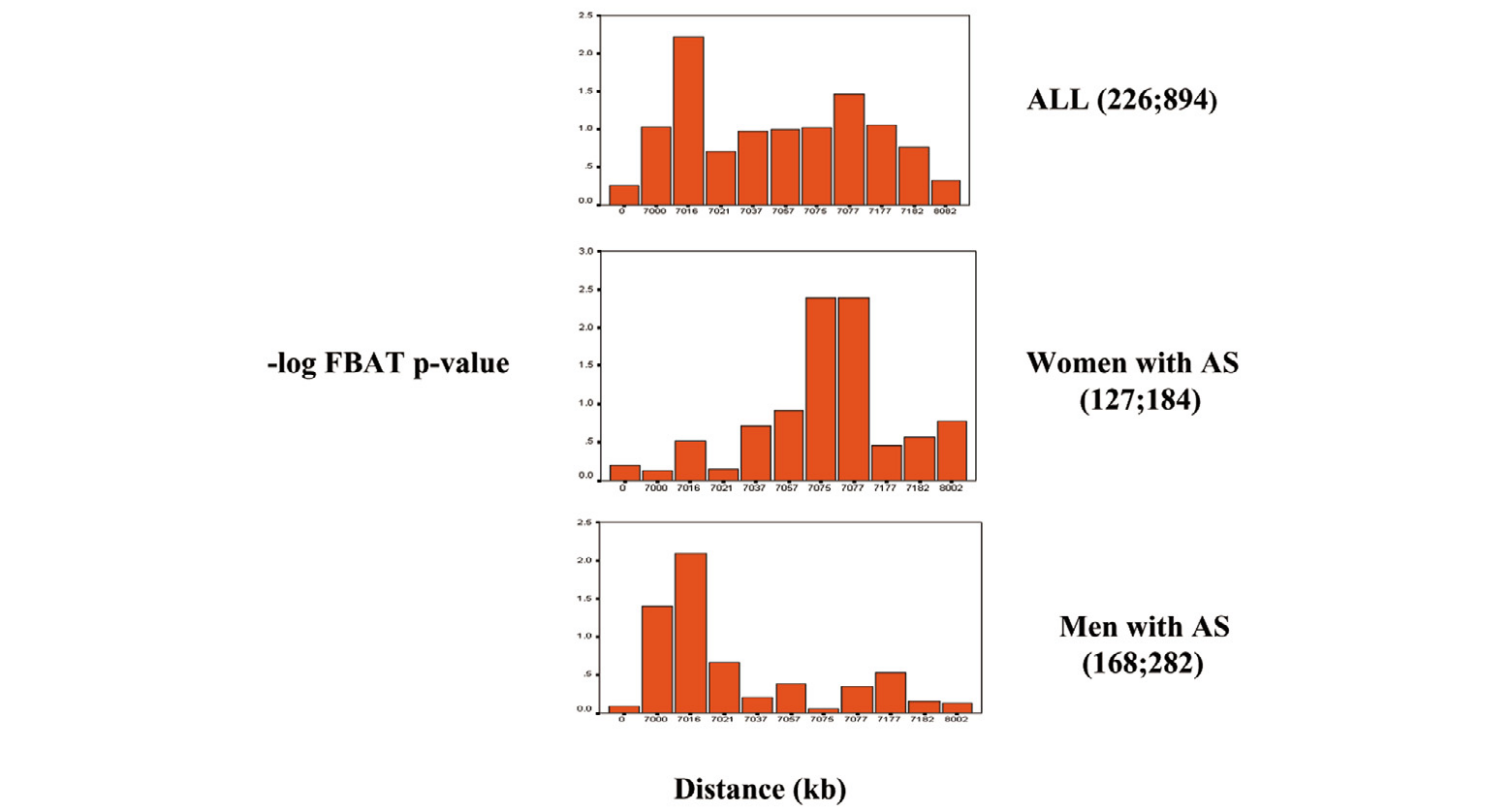
A summary of family-based association analyses (FBAT; additive model and biallelic tests). Each column in the charts represents the -log FBAT *P *value for each marker located at a distance relative to one another. *D5S1953 *is positioned at 0 distance. Chart ALL shows analysis of 226 ankylosing spondylitis (AS) nuclear families with 894 persons. Chart Women shows analysis of 127 AS families with 184 affected women. Chart Men shows analysis of 168 AS families with 282 affected men.

**Table 1:** Characteristics of 226 nuclear families included in the family-based association studies

Number of affected siblings	Number of unaffected siblings	Number of unaffected parents	Number of affected parents	Number of nuclear families
2	0	0	0	71
2	0	2	0	33
2	0	1	0	14
2	0	1	1	5
2	1	0	0	4
2	2	0	0	3
2	1	0	1	3
2	1	2	0	2
2	2	2	0	1
3	0	0	0	9
3	0	2	0	6
3	0	1	0	4
3	0	1	1	2
3	1	0	0	1
3	1	1	0	1
4	0	0	0	1
4	0	2	0	2
4	0	1	0	1
4	1	2	0	1
1	0	2	0	25
1	0	1	1	8
1	0	1	0	2
1	0	0	1	7
1	1	0	0	3
1	1	0	1	3
1	1	2	0	1
1	1	1	1	1
1	2	0	0	1
1	2	0	1	1
1	2	2	0	1
1	3	0	0	1
1	3	2	0	1
0	2	0	0	1
0	2	1	1	2
0	1	0	1	3

**Table 2:** Gender information for affected individuals in the 201 ankylosing spondylitis families

Number of affected men/women in a family	Number of families
Families with both affected men and women	94

1/1	60
2/1	17
3/1	1
1/2	8
2/2	3
3/2	1
1/3	2
2/3	2
Families with only affected men	74
1/0	5
2/0	54
3/0	12
4/0	2
5/0	1
Families with only affected women	33
0/1	2
0/2	26
0/3	4
0/4	1

**Table 3:** FBAT-e analyses conducted in 226 ankylosing spondylitis nuclear families (201 pedigrees, 894 persons)

Marker	Allele	Allele frequency	Number of informative families	Z score	*P*
Additive model: biallelic test					

*D5S1953*	2	0.45	54	0.57	0.569
*rs26307*	C	0.81	35	1.73	0.084
*rs27356*	C	0.80	39	2.54	0.011*
*3088132*	G	0.79	28	1.28	0.198
*rs153929*	A	0.76	48	1.61	0.107
*rs258215*	A	0.59	43	1.58	0.113
*rs28006*	C	0.74	34	1.62	0.104
*rs25957*	C	0.76	36	2.02	0.043*
*ANKH-OR*	1	0.47	59	1.62	0.105
*D5S1991*	2	0.48	58	1.33	0.181
*D5S1954*	1	0.64	54	0.65	0.515

Recessive model: biallelic test					

*D5S1953*	2	0.43	48	0.45	0.683
*rs26307*	C	0.80	40	1.49	0.137
*rs27356*	C	0.79	44	2.32	0.020*
*3088132*	G	0.79	31	1.19	0.233
*rs153929*	A	0.76	50	1.86	0.062
*rs258215*	A	0.58	32	1.55	0.120
*rs28006*	C	0.74	37	1.46	0.142
*rs25957*	C	0.76	38	1.65	0.098
*ANKH-OR*	2	0.52	37	-2.20	0.027*
*D5S1991*	1	0.52	45	-1.96	0.050*
*D5S1954*	1	0.64	54	0.65	0.517

*Statistically significant findings. FBAT, family-based association testing.

**Table 4:** FBAT-e analyses using setafftrait 0 -1 0, testing specifically for affected women

Marker	Allele	Allele frequency	Number of informative families	Z score	*P*
Additive model: biallelic test					

*D5S1953*	1	0.56	47	0.47	0.635
*rs26307*	T	0.19	22	0.32	0.751
*rs27356*	T	0.20	24	1.03	0.302
*3088132*	C	0.21	20	0.37	0.712
*rs153929*	G	0.24	46	1.31	0.191
*rs258215*	T	0.41	30	1.54	0.123
*rs28006*	T	0.26	31	2.82	0.004*
*rs25957*	G	0.23	23	2.82	0.004*
*ANKH-OR*	2	0.52	59	0.94	0.347
*D5S1991*	1	0.52	53	1.10	0.270
*D5S1954*	2	0.36	45	1.38	0.168

Recessive model: biallelic test					

*D5S1953*	2	0.44	39	-1.21	0.227
*rs26307*	C	0.81	26	0.52	0.606
*rs27356*	T	0.20	10	1.81	0.069
*3088132*	G	0.79	22	0.08	0.930
*rs153929*	A	0.76	44	-1.39	0.162
*rs258215*	A	0.58	21	-1.76	0.077
*rs28006*	C	0.74	28	-2.49	0.012*
*rs25957*	C	0.76	23	-2.25	0.024*
*ANKH-OR*	2	0.52	34	1.14	0.254
*D5S1991*	1	0.51	33	1.52	0.127
*D5S1954*	1	0.64	45	-1.54	0.122

*Statistically significant findings. FBAT, family-based association testing.

**Table 5:** FBAT-e analyses using setafftrait 1 0 0, testing specifically for affected men

Marker	Allele	Allele frequency	Number of informative families	Z score	*P*
Additive model: biallelic test					

*D5S1953*	2	0.44	47	0.25	0.805
*rs26307*	C	0.80	22	2.06	0.039*
*rs27356*	C	0.79	25	2.63	0.008*
*3088132*	G	0.79	18	1.23	0.216
*rs153929*	A	0.76	36	0.49	0.619
*rs258215*	A	0.58	32	0.81	0.419
*rs28006*	T	0.26	37	0.15	0.877
*rs25957*	C	0.77	33	0.76	0.446
*ANKH-OR*	1	0.48	64	1.04	0.297
*D5S1991*	2	0.49	64	0.39	0.696
*D5S1954*	2	0.36	49	0.33	0.736

Recessive model: biallelic test					

*D5S1953*	1	0.56	40	-0.83	0.406
*rs26307*	C	0.81	25	2.51	0.012*
*rs27356*	C	0.80	28	2.99	0.002*
*3088132*	G	0.79	22	1.44	0.149
*rs153929*	A	0.76	39	0.71	0.477
*rs258215*	A	0.59	23	0.60	0.543
*rs28006*	T	0.26	16	0.33	0.739
*rs25957*	C	0.76	29	0.64	0.518
*ANKH-OR*	2	0.52	44	-1.26	0.205
*D5S1991*	1	0.51	46	-0.71	0.472
*D5S1954*	1	0.64	43	-0.65	0.510

*Statistically significant findings. FBAT, family-based association testing.

**Table 6:** HBAT-e analyses using *ANKH *markers in four haplotype blocks defined in HapMap

Markers in the haplotype block	AS nuclear families (n = 226)	Testing specifically for affected women	Testing specifically for affected men
Additive model			
*rs26307*, *rs27356*	[C,C]; 0.78; 39; 0.04	NS	[C,C]; 0.79; 24; 0.014
*3088132*, *rs153929*	NS	NS	NS
*rs28006*, *rs25957*	NS	[T,G]; 0.25; 19; 0.007	NS
*ANKH-OR*, *D5S1991*	[1,2]; 0.43; 57; 0.013	NS	NS
Recessive/dominant model			
*rs26307*, *rs27356*	[C,C]; 0.78; 40; 0.02	NS	[C,C]; 0.79; 25; 0.004*
*3088132*, *rs153929*	[G,A]; 0.70; 32; 0.02	NS	NS
*rs28006*, *rs25957*	NS	[C,C]; 0.71; 18; 0.004*	NS
*ANKH-OR*, *D5S1991*	NS	NS	NS

Data are expressed as [allele]; allele frequency; number of informative families; *P *value.

*Significant *P *value after Bonferroni correction. (Because eight tests [four haplotypes and two models] were carried out in the haplotype-based association testing [HBAT]-e analyses, *P *< 0.00625 [0.05/8] is considered statistically significant.) AS, ankylosing spondylitis; NS, not significant.

**Table 7:** FBAT-e analyses considering 226 ankylosing spondylitis nuclear families (201 pedigrees, 894 persons): summary of results using setafftrait 1 -1 0

Marker	Allele	Allele frequency	Number of informative families	Z score	*P*
Additive model: biallelic test					

*D5S1953*	1	0.56	63	0.15	0.879
*rs26307*	C	0.81	35	1.29	0.195
*rs27356*	C	0.80	39	1.12	0.261
*3088132*	G	0.79	29	0.69	0.489
*rs153929*	G	0.24	52	0.61	0.541
*rs258215*	T	0.41	41	0.46	0.638
*rs28006*	T	0.26	44	1.69	0.090
*rs25957*	G	0.23	39	0.90	0.366
*ANKH-OR*	1	0.48	80	0.20	0.841
*D5S1991*	1	0.52	80	0.29	0.769
*D5S1954*	2	0.36	63	1.21	0.227

Recessive model: biallelic test					

*D5S1953*	1	0.56	46	-0.89	0.370
*rs26307*	C	0.81	37	2.17	0.030*
*rs27356*	C	0.80	41	1.91	0.055
*3088132*	G	0.79	29	1.17	0.240
*rs153929*	G	0.24	18	0.55	0.582
*rs258215*	A	0.59	29	-0.75	0.453
*rs28006*	C	0.74	40	-1.13	0.186
*rs25957*	C	0.77	36	-0.66	0.507
*ANKH-OR*	2	0.52	50	-0.34	0.733
*D5S1991*	2	0.49	52	-0.28	0.779
*D5S1954*	1	0.64	61	-1.53	0.126

*Statistically significant findings. FBAT, family-based association testing.

**Table 8:** FBAT-e analyses considering 108 ankylosing spondylitis nuclear families (94 pedigrees, 425 persons) in which both affected men and women are present in each family: summary of the results using setafftrait 1 -1 0

Marker	Allele	Allele frequency	Number of informative families	Z score	*P*
Additive model: biallelic test					

*D5S1953*	1	0.56	39	0.77	0.437
*rs26307*	C	0.90	10	0.50	0.617
*rs27356*	C	0.77	17	0.15	0.875
*3088132*	C	0.21	13	0.63	0.527
*rs153929*	G	0.23	32	0.81	0.418
*rs258215*	T	0.49	21	1.62	0.104
*rs28006*	T	0.29	27	2.81	0.004*
*rs25957*	G	0.32	22	2.37	0.017*
*ANKH-OR*	1	0.48	51	0.25	0.801
*D5S1991*	2	0.50	48	0.13	0.891
*D5S1954*	2	0.33	31	1.13	0.254

Recessive model: biallelic test					

*D5S1953*	2	0.44	31	-1.34	0.177
*rs26307*	C	0.79	19	1.00	0.314
*rs27356*	C	0.80	20	1.17	0.239
*3088132*	G	0.79	14	-0.26	0.788
*rs153929*	G	0.23	10	1.06	0.288
*rs258215*	A	0.51	12	-2.49	0.012*
*rs28006*	C	0.71	20	-2.344	0.019*
*rs25957*	C	0.67	15	-1.97	0.048*
*ANKH-OR*	1	0.48	27	0.83	0.406
*D5S1991*	2	0.49	29	0.98	0.322
*D5S1954*	1	0.66	31	-1.67	0.093

*Statistically significant findings. FBAT, family-based association testing.
